# Applied novel functionality in separation procedure from leaching solution of zinc plant residue by using non-aqueous solvent extraction

**DOI:** 10.1038/s41598-023-27646-9

**Published:** 2023-01-20

**Authors:** Fatemeh Badihi, Ali Haghighi Asl, Mehdi Asadollahzadeh, Rezvan Torkaman

**Affiliations:** 1 Faculty of Chemical, Gas and Petroleum Engineering, Semnan University, Semnan, 35195-363 Iran; 2 Nuclear Fuel Cycle Research School, Nuclear Science and Technology Research Institute, Tehran, 11365-8486 Iran

**Keywords:** Engineering, Chemical engineering

## Abstract

Traditional solvent extraction (SX) procedures limit metal separation and purification, which consist of the organic and aqueous phases. Because differences in metal ion solvation lead to distinct distribution properties, non-aqueous solvent extraction (NASX) considerably expands the scope of solvent extraction by replacing the aqueous phase with alternate polar solvents. In this study, an experimental design approach used non-aqueous solvent extraction to extract cobalt from zinc plant residue. The aqueous phase comprises ethylene glycol (EG), LiCl and metal ions. In kerosene, D2EHPA, Cyanex272, Cyanex301, and Cyanex302 extractants were used as a less polar organic phase. Various factors were investigated to see how they affected extraction, including solvent type, extractant type and phase ratio, pH, Co(II) concentration, and temperature. The results revealed that at a concentration of 0.05 M, the Cyanex301 extractant could achieve the requisite extraction efficiency in kerosene. The optimal conditions were chosen as the concentration of Cyanex 301 (0.05 M), the concentration of cobalt (833 ppm), the pH (3.5), and the percent of EG (80%). As a result, during the leaching process, these systems are advised for extracting and separating a combination of various metal ions.

## Introduction

Metal utilization increases in developed industries. As a result, natural resources for metal production are depleted and a considerable amount of waste is generated, resulting in financial losses and environmental concerns^1–5^. Cobalt is a mineral found in soil, plants, water, animals, and is one of the vitamin B12 components. Cobalt deficiency leads to developmental delays, anemia, and a loss of appetite^6^. Although cobalt is regularly combined with copper, zinc, nickel, and other metals, it is primarily employed in producing lithium and nickel-metal hydride batteries^7^. Mobile phones, tablets, PCs, and other electronic gadgets use these batteries. Lithium batteries are chosen over nickel-metal hydride batteries because of their low weight, small size, and high voltage^8,9^.

Cobalt recycling from lithium-ion batteries is both ecologically benign and cost-effective due to its limited availability and widespread applications^10,11^. Cobalt demand has risen dramatically in recent years due to its extensive use. Slags and tailings from metallurgical activities may have cobalt-bearing waste with recycling potential^9,12–14^. Many strategies may be mentioned to recover cobalt from industrial effluents, including solvent extraction, chemical precipitation, ion exchange, bio-sorption, membrane processes, and aqueous two-phase systems^15–17^. Specific methods have been employed in the past to separate and purify various metals from primary and secondary resources^18–21^. Due to its remarkable selectivity and significant cost savings, solvent extraction is one of the most commonly used procedures in hydrometallurgy and on an industrial scale to separate and purify cobalt ions^22–25^. On the other hand, traditional solvent extraction necessitates numerous phases involving huge amounts of acids, resulting in large volumes of aqueous acidic solutions that must be handled^24–28^.

Solvometallurgy is a new branch of extractive metallurgy in which non-aqueous solvent extraction is comparable to hydrometallurgy and has been developed by researchers to replace traditional solvent extraction by employing polar organic solvents rather than aqueous solutions^26–28^. Compared to conventional solvent extraction with an aqueous feed phase, non-aqueous solvent extraction has some advantages. Extraction from a non-aqueous solution may have a different mechanism than extraction from an aqueous solution, and this difference could be used to design novel, highly selective separation techniques^29^. Metal ions or metal complexes with a high tendency to hydrolyze in water can also be extracted via non-aqueous solvent extraction^27,30^. The solvent pairs for non-aqueous solvent extraction must meet the following criteria: (1) formation of two phases upon mixing and low mutual solubility of the solvents; (2) rapid phase separation after mixing (= short phase disengagement time); (3) the extractant must be soluble in the less polar phase; (4) the starting metal compounds or salts must be soluble in the more polar phase and the extracted metal complexes in the less polar phase; (5) the starting metal compounds or salts must be soluble. Because many organic solvents are mutually miscible, the first requirement is the most difficult to meet^31^.

Batchu et al. investigated the extraction of Nd (III) and Dy (III) from aqueous nitrate solutions and non-aqueous ethylene glycol. They discovered ethylene glycol solutions had more prominent separation factor than water solutions. The extraction efficiency for Cyanex923, Cyanex272, and tributyl phosphate was obtained at 90%, 35% and 20%, respectively. The extraction efficiency with a non-aqueous system (90%) was higher than the aqueous system (70%) with Cyanex923 (0.1 M)^30^. In another study, the separation of Y(III) and Eu(III) was investigated with a pure ethylene glycol system and LiCl (3 M) into the organic phase, including Cyanex923, 10 vol% 1-decanol, diluted in GS190. The data showed that yttrium was separated from europium from EG feed solutions (separation factor = 46), which is not possible from aqueous feed solutions (separation factor = 1)^26^. Li and colleagues looked into the extraction of cobalt and samarium. They showed that using a polar organic solvent instead of water improved metal separation. They extracted cobalt and samarium chlorides from aqueous and ethylene glycol solutions, respectively, using trioctylmethylammonium chloride (Aliquate 336) diluted in toluene and lithium chloride as a chloride source. According to the researchers, cobalt and samarium were separated from the water phase, whereas cobalt was removed more efficiently from ethylene glycol phases. At LiCl concentration equal to 3.0 M, 73.9% of Co(II) was extracted from the aqueous solution, while over 99% was obtained from the EG solution. Also, the best result with a separation factor equal to 3971 was obtained for the separation of Co(II) from Ni(II) at 3.0 M LiCl and EG system^32^. Dewulf and co-workers illustrated the extraction of rare earth elements from non-aqueous systems with different solvents. The results showed that both contact-ion-pair formation and solvation strength was the main parameters in the extraction procedure^33^. In the study of Binnemans and co-workers, the solvometallurgical approach was investigated for the separation of indium(III) and zinc(II). The mixture of ionic liquid and Aliquate 336 was used as the extractant phase. The extraction procedure with 98% efficiency was obtained in the mixer-settler system^34^. In another study, the development flowsheet was illustrated for the extraction of heavy rare earth elements with non-aqueous solvent extraction with high efficiency higher than 99%^35^.

El-Hefny and co-workers illustrated the separation of gadolinium and neodymium with a non-aqueous solvent extraction system. The results showed the high separation factor ~ 22 was obtained with EG = 50%, [LiNO_3_] = 1.0 mol/L, [Cyanex572] = 0.0175 mol/L, Equilibration time = 15 min^22^.

It is observed that studies of the solvometallurgy process for cobalt extraction are scarce. This study focuses on optimizing effective parameters on the cobalt recovery from zinc plant residue using responses surface methodology with a new extraction procedure with a non-aqueous system and Cyanex301 as an extractant. The justification for performing a central composite approach on the parameters utilized in the current investigation is that a second-order model could be developed to extract cobalt ions. A survey of the interacting effects of experimental parameters with this technique has not been reported elsewhere.

## Experimental

### Materials

The salt of CoCl_2_.6H_2_O (Merck company, 98.0%) was acquired for the preparation of cobalt solution. Bis (2,4,4-trimethylpentyl monothiophosphinic acid) (Cyanex302) (Aldrich company, 90.0%), bis(2,4,4-trimethylpentyl)dithiophosphinic acid (Cyanex301) (Aldrich company, 90.0%), bis-(2,4,4-trimethylpentyl) phosphinic acid (Cyanex272) (Aldrich company, 90.0%), and Di-(2-ethylhexyl)phosphoric acid (D2EHPA) (Merck company, 95.0%), as the extractants and kerosene (99.9%) as the diluted phase from Merck company, were used in the experiments. Ethylene glycol from Sigma Aldrich, 99.5% was used as the non-aqueous system. Another diluted phase such as, chloroform (99.8%), 1-decanol (99.0%), and toluene (99.9%) was provided from Merck. HCl (37.0%, Merck) and NaOH (99.9%, Merck) solutions were used to reach the appropriate pH of solutions in the experiments. As reverse extraction agents, acids like sulfuric acid (96.0%, Merck), nitric acid (65.0%, Merck), and hydrochloric acid were utilized. Ascorbic acid (99.0%, Merck), ammonium thiocyanate (99.0%, Merck), acetone (99.0%, Merck), and hydrochloric acid were used as cobalt detectors using a UV spectrophotometer.

### The non-aqueous solvent extraction (NASX) procedure

**Figure 1:**
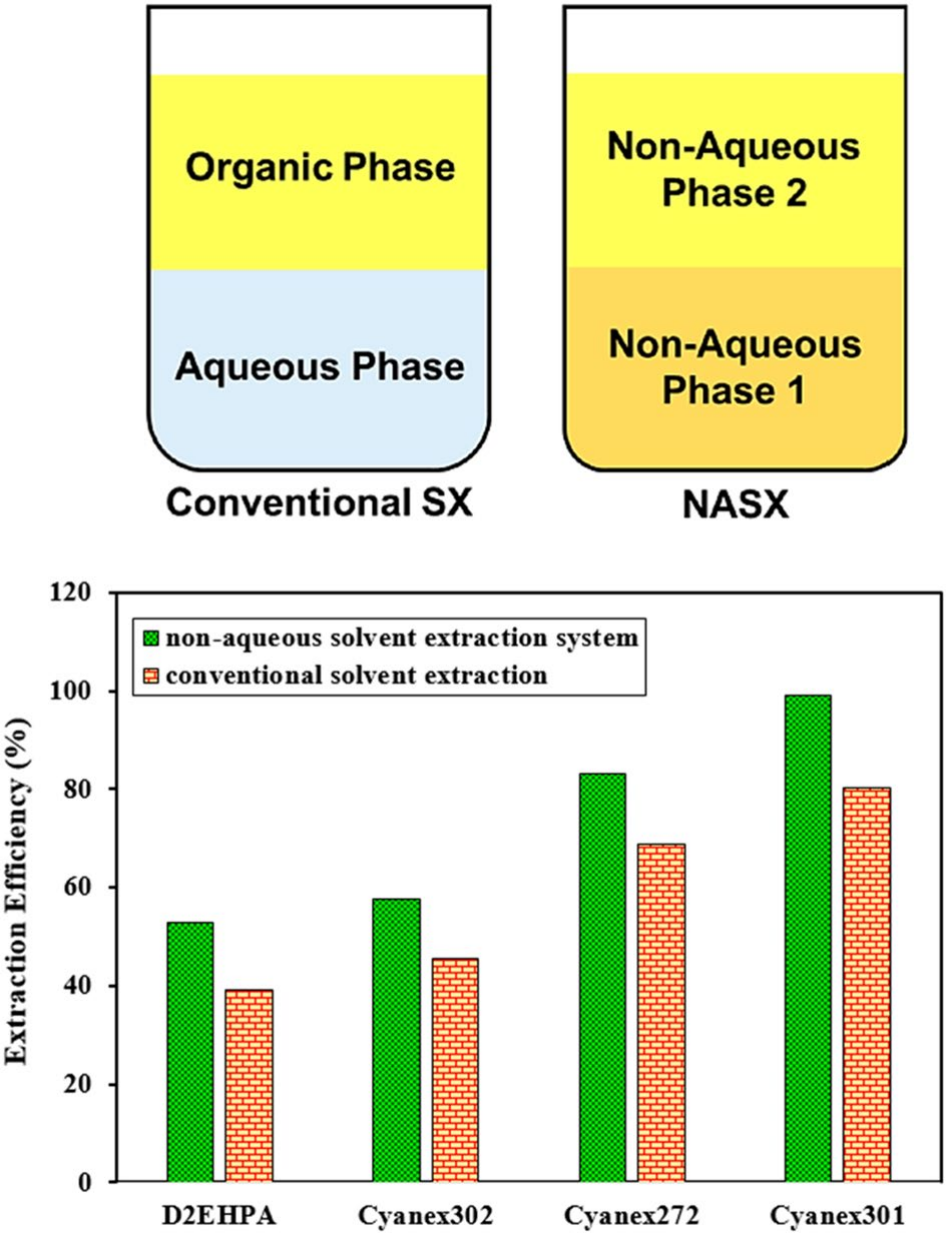
Comparison of (left) conventional solvent extraction (SX) and non-aqueous solvent extraction (NASX) systems [(Co(II)) = 700 ppm; 80%EG + 20%Water in NASX and 100% Water in SX; pH = 3; (LiCl) = 1 M; 0.05 M concentration for all extractants].

Figure 1 shows a typical solvent extraction (SX) system consisting of an organic phase and an aqueous phase, and a non-aqueous solvent extraction system consisting of two non-aqueous phases.

For non-aqueous solvent extraction tests, equal volumes of the less polar organic phase (Cyanex301 in kerosene) and more polar organic phase (metal ions in ethylene glycol + lithium chloride) are combined in 15 ml test tubes. The mixture was agitated for 10 min and was centrifuged at 6000 rpm for 10 min to separate the phases. All of the samples were measured twice. The fraction of removed metal ions or extraction efficiency (%E) was calculated using the following equation:

1 $${{\% E}} = \frac{{\left( {\text{C}} \right)_{{{\text{LP}}}} }}{{\left( {\text{C}} \right)_{{{\text{Tot}}}} }} \times 100$$

where, (C)_Tot_ is the overall solution concentration and (C)_LP_ is the metal concentration in the less polar phase.

The kerosene phase (Cyanx301) was charged with 800 ppm cobalt from an ethylene glycol (+ LiCl) solution. Sulfuric acid at different concentrations was used to isolate the pregnant phase. The following equation is used to determine the percentage of stripping:

2 $${{\% S}} = \frac{{\left[ {\text{M}} \right]{\text{aq}},{\text{a}}}}{{\left[ {\text{M}} \right]{\text{org}},{\text{t}}}} \times 100$$

where, [M]_aq,a_ represents the metal ion’s equilibrium concentration in the stripping acid and [M]_org,t_ represents the metal ion’s starting concentration in the loaded organic phase, respectively.

### Optimization using RSM approach

This work employed the response surface test design to evaluate the effects of polyethylene glycol content, cobalt feed solution concentration, Cyanex301 concentration, pH of a solution and their interactions. The parameters and levels chosen are listed in Table 1.

**Table 1:** Parameters and selected upper and lower levels with central composite design.

Parameter type	Low level	High level
[Cyanex301]	0.005	0.05
pH	0	6
%EG	50	90
[Co]	600	1000

## Results and discussion

Because solvent extraction necessitates the coexistence of two organic phases, the solubility of two phases must be established. The findings imply that combining ethylene glycol and kerosene is an excellent choice for extracting non-aqueous solvents because when equal amounts of both solvents are combined, two phases are formed with no perceptible volume change. The optimal point was chosen once all of the experimental design experiments were completed, and all subsequent experiments were carried out at this point. The comparison of the extraction procedure with typical solvent extraction (SX) and a non-aqueous solvent extraction system (NASX) for the extraction of cobalt ions (700 ppm) with different extractants (0.05 M concentration) is shown in Fig. 1. The results showed that the NASX system containing 20%water + 80%EG was better than the SX system including 100% water with no ethylene glycol. Therefore, the other experiments were carried out with the NASX systems, that the optimized conditions were reported in the following sections.

### Effect of extractant type on non-aqueous solvent extraction

**Figure 2:**
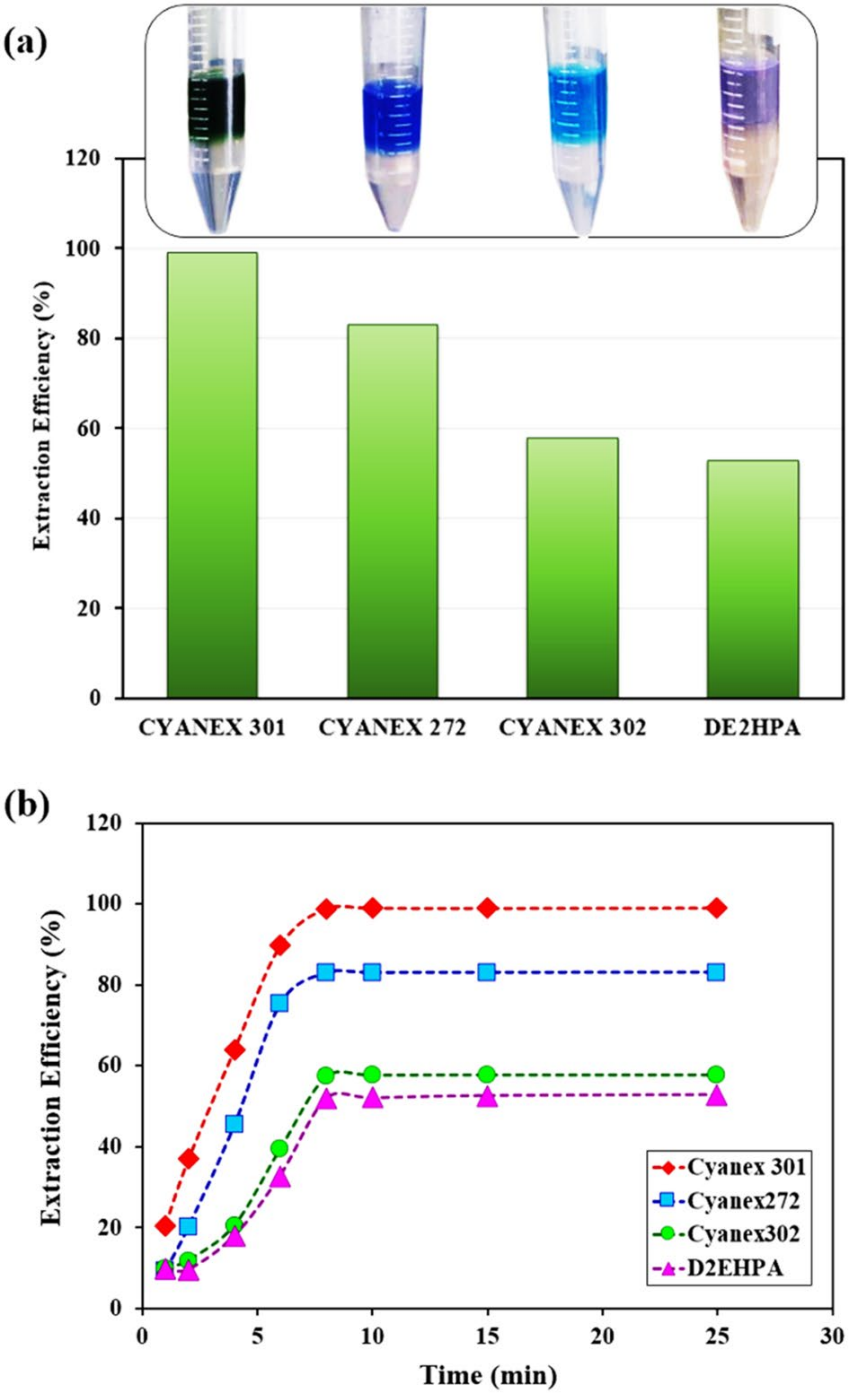
(**a**) Effect of extractant type on the extraction efficiency in non-aqueous solvent extraction system for Co(II) removal; (**b**) Effect of time on the extraction efficiency [(Co(II)) = 700 ppm; 80%EG + 20%Water; pH = 3; [LiCl] = 1 M; 0.05 M concentration for all extractants].

The first step in considering an extraction system is finding the appropriate extractant type to recover cobalt ions. The results of the different extractants in Fig. 2a showed that the Cyanex301 extractant is more desirable in the non-aqueous solvent system and can be utilized in cobalt ion recovery at pH intervals of 3 to 7 with a high percentage. The color change of each extractant in the separation of cobalt ions is also shown in this diagram, which changes the sludge from green to bold blue, light blue, and purple with Cyanex301, Cyanex272, Cyanex302, and D2EHPA, respectively.

Figure 2b depicts the percent extraction of cobalt ions versus the time to reach the equilibrium with each extractant. It was evident from the results that the extraction percentage increments as the extraction period is increased from 1 to 6 min; after this time, the extraction percent reaches maximum values and no significant variation is observed after 8 min. Therefore, the 10 min equilibrium time was chosen as the best time to reach the maximum extraction.

### Results from experimental design approach

Table 2 shows the experimental design of different parameters along with the experimental data. The highest percentage of cobalt removal (99.89%) was obtained at pH 3, cobalt concentration 800 ppm, ethylene glycol 70% and Cyanex301 concentration 0.05 M. While the lowest removal percentage (17.58%) was observed at pH 0, the Cyanex301 concentration 0.028 M, cobalt concentration 800 ppm, and ethylene glycol 70%.

**Table 2:** Conditions of thirty designed experiments with obtained results.

Run	Cyanex 301 concentration (M)	pH	% Ethylene Glycol	Co(II)	%E	%E
				Concentration (ppm)	Actual data	Predicted data
1	0.016	1.5	80	700	37.08	42.81
2	0.028	3	50	800	39.38	53.02
3	0.028	3	70	800	53.48	62.12
4	0.028	3	70	800	69.40	62.12
5	0.016	4.5	80	700	46.35	50.62
6	0.028	3	70	800	60.23	62.12
7	0.016	4.5	60	700	41.05	42.34
8	0.028	3	70	800	59.45	62.12
9	0.028	3	70	800	62.79	62.12
10	0.028	6	70	800	51.36	46.14
11	0.016	1.5	60	900	38.74	32.29
12	0.039	1.5	60	900	68.51	62.21
13	0.039	4.5	80	700	90.90	97.07
14	0.016	1.5	80	900	40.73	38.71
15	0.016	4.5	60	900	34.29	31.56
16	0.039	1.5	80	700	75.05	76.55
17	0.016	4.5	80	900	28.91	34.02
18	0.039	4.5	80	900	80.01	81.00
19	0.028	3	70	800	66.67	62.12
20	0.039	4.5	60	900	81.01	74.20
21	0.028	0	70	800	17.58	25.83
22	0.039	1.5	80	900	75.14	72.98
23	0.039	4.5	60	700	83.87	84.44
24	0.005	3	70	800	30.96	28.79
25	0.028	3	90	800	82.84	72.24
26	0.05	3	70	800	99.89	105.12
27	0.016	1.5	60	700	32.84	30.56
28	0.028	3	70	1000	47.73	57.31
29	0.039	1.5	60	700	66.10	59.96
30	0.028	3	70	600	78.18	71.64

In the prediction of the best model, the R^2^ for the removal of metal ions values of quadratic and cubic models were high (> 0.9) compared to the other models. However, the Design Expert expressed that the cubic model was aliased, and suggested the quadratic model instead. The aliased model could not be acknowledged and selected as the best model because it dismisses all the points contributing to the highest value of R^2^. Therefore, the quadratic model was selected as the best model (R^2^ 0.9210, Adjusted R^2^ 0.8473) to fit all the experimental data collected to remove Co(II). To build a link between the independent variables and the extraction (response) %, the response level approach can be employed; the resulting extraction percentage model is as follows:

3 $$\begin{aligned} {{\% E}} = & - 82.164 - 257.426 \times {\text{A}} + 36.824 \times {\text{B}} + 1.395 \times {\text{C}} + 0.031 \times {\text{D}} \\ & + 188.291 \times {\text{A}} \times {\text{B}} - 9.666 \times {\text{A}} \times {\text{C}} + 0.117 \times {\text{A}} \times {\text{D}} - 0.066 \times {\text{B}} \times {\text{C}} \\ & - 0.021 \times {\text{B}} \times {\text{D}} - 0.0014 \times {\text{C}} \times {\text{D}} + 2311239.32 \times A^{2} \\ & - 2.903 \times B^{2} + 0.0013 \times C^{2} + 0.00006 \times D^{2} \\ \end{aligned}$$

In the above equation, A, B, C, and D were denoted to Cyanex301 concentration, pH of the aqueous solution, ethylene glycol percentage, and cobalt concentration, respectively.

A summary of model fits analysis (variance analysis) on the removal of Co(II) is shown in Table 3. The model was selected based on the highest value of F (12.49) and the highest value of correlation (R^2^ ~ 0.921). *P* value is a statistical value to test the level of significance of the model. To remove Co(II), the *P* values for the mismatch test were lower than 0.0001, indicating that the quadratic model has the best model in the prediction of data. The Lack of Fit *F* value of 2.72 implies the Lack of Fit is not significant relative to the pure error. There is a 14.04% chance that a Lack of Fit *F* value this large could occur due to noise.

**Table 3:** Analysis of variance (ANOVA) data.

Source	Sum of squares	df	Mean Square	*F* value	*p* value
**Model**	11,967.55	14	854.82	12.49	< 0.0001
A-[cyanex 301]	8741.95	1	8741.95	127.76	< 0.0001
B-pH	584.11	1	584.11	8.54	0.0105
C-% EG	542.97	1	542.97	7.94	0.0130
D-[Co]	314.11	1	314.11	4.59	0.0490
AB	161.62	1	161.62	2.36	0.1452
AC	18.93	1	18.93	0.2766	0.6066
AD	0.2796	1	0.2796	0.0041	0.9499
BC	15.71	1	15.71	0.2296	0.6387
BD	156.26	1	156.26	2.28	0.1515
CD	33.91	1	33.91	0.4956	0.4922
A^2^	55.41	1	55.41	0.8098	0.3824
B^2^	1170.53	1	1170.53	17.11	0.0009
C^2^	0.4437	1	0.4437	0.0065	0.9369
D^2^	9.48	1	9.48	0.1386	0.7149
**Residual**	1026.41	15	68.43		
Lack of fit	867.07	10	86.71	2.72	0.1404
Pure error	159.34	5	31.87		
**Cor total**	12,993.95	29			

**Figure 3:**
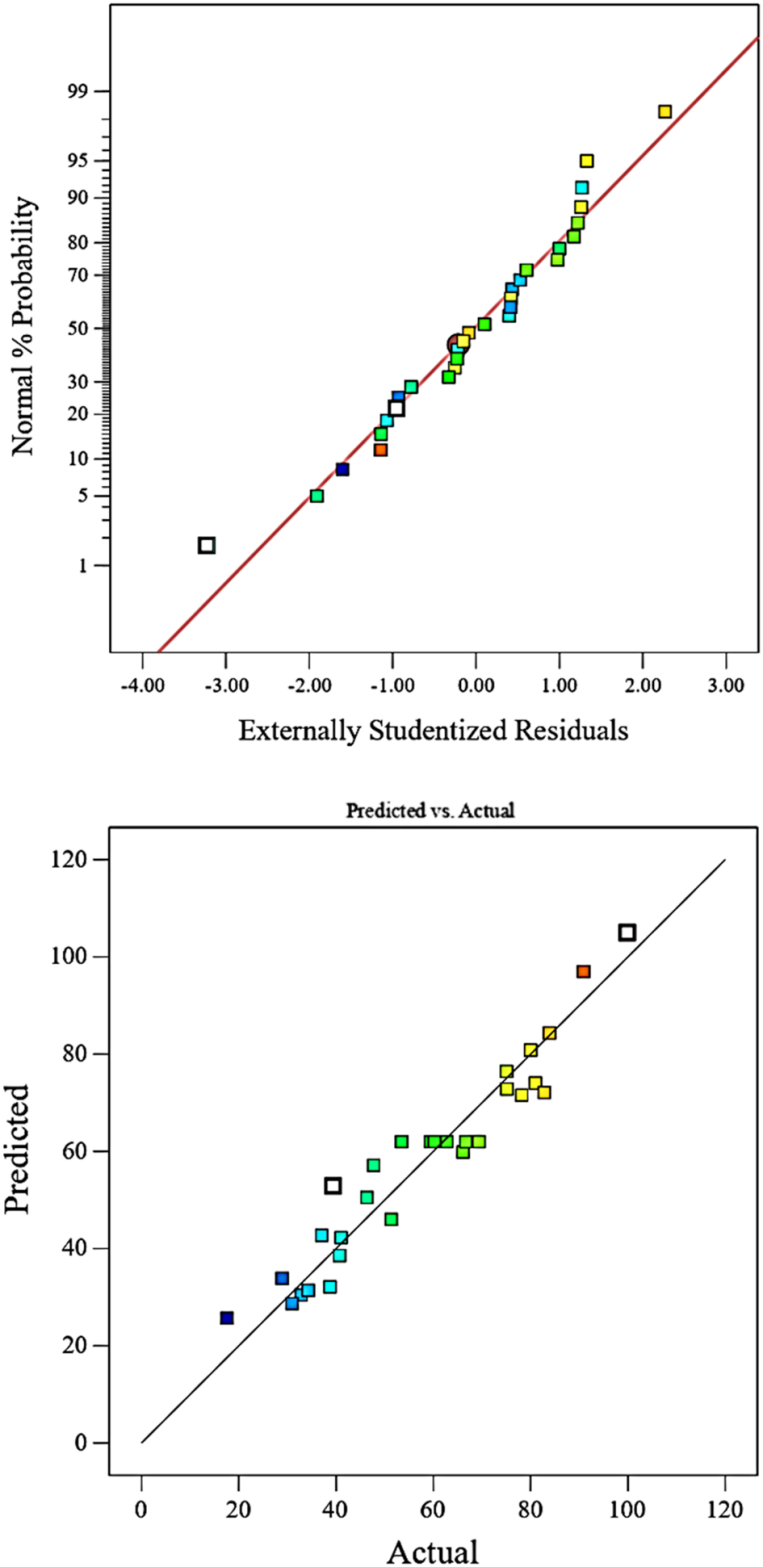
Normal plot and the prediction plot with the deviation from the actual data.

In Fig. 3, a normal distribution of the residuals is shown using a normal plot. It is observed that points do not have a specific shape and are around the 45-degree line and this plot confirms the model. Also, the values obtained from the model are compared with the actual data. The results are almost along the 45-degree line and this means that the predicted values are very close to the experimental values and the validation of the model is confirmed with this plot.

The validation of the quadratic model was carried out with the different variables and the determination of new responses with the above equation. A comparison of results predicted by the mathematical model and experimental results is shown in Table 4. As can be seen, the predicted and experimental findings are highly correlated and the error between the model and practical data is very low (< 10%).

**Table 4:** Comparison of the correlation of the predicted results with the experimental results.

Observation	Response of experimental (%)	Response of model (%)	Error (%)
[Cyanex301] = 0.01 pH = 2 %EG = 75 [Co] = 650	41.69	39.01	2.68
[Cyanex301] = 0.04 pH = 3.5 %EG = 55 [Co] = 750	82.78	79.45	3.33
[Cyanex301] = 0.048 pH = 4 %EG = 85 [Co] = 850	83.10	90.81	7.72

#### Evaluation of 3D plots for obtained data

**Figure 4:**
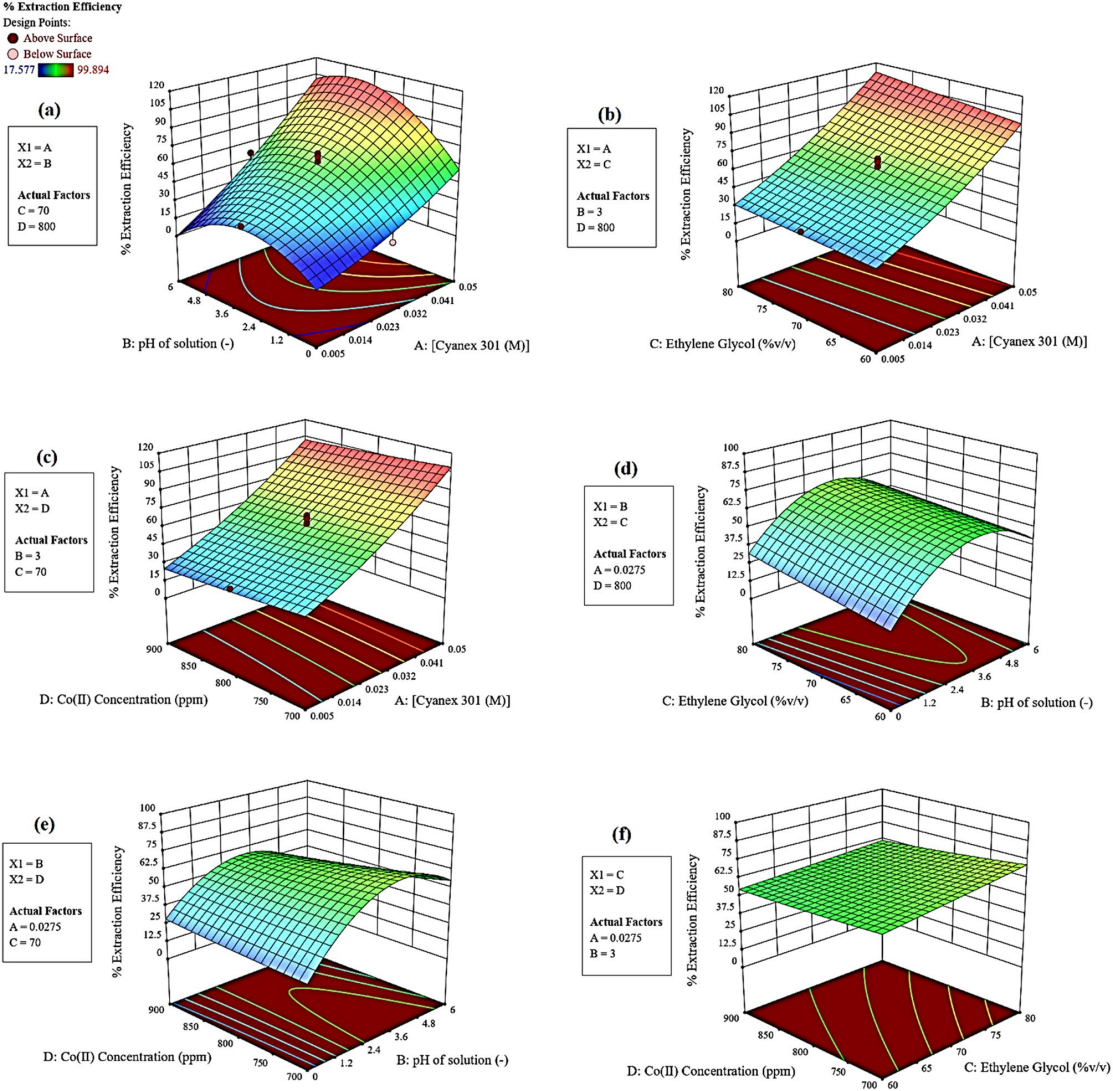
3D plots for the description of each parameter on the Co(II) removal under non-aqueous system; (**a**) effect of pH and Cyanex301 concentration; (**b**) effect of ethylene glycol percent and Cyanex301 concentration; (**c**) effect of cobalt concentration and Cyanex301 concentration; (**d**) effect of ethylene glycol percent and pH; (**e**) effect of cobalt concentration and pH; (**f**) effect of cobalt concentration and ethylene glycol percent.

The effect of pH and concentration of the primary extractant concentration in kerosene was investigated in Fig. 4a. The effects of pH changes in the range 0 to 6 were studied. As shown in this figure, increasing the pH improves the extraction efficiency due to the tendency of the reaction to the complex formation to the organic phase with the acidic extractant. The optimal pH of 3.5 was obtained with the experimental data. After this data, the variation on the results with increasing behavior is very low. The extractant concentration was tested in the range of 0.005 to 0.05 M and the optimal concentration of Cyanex301 was considered 0.05 M. The more values of extractant lead to the appropriate condition for the extraction of cobalt ions.

The effect of Cyanex301 concentration in the range of 0.005–0.05 M and the percentage of ethylene glycol in the range of 50 to 90% in an aqueous system was investigated. As shown in Fig. 4b, increasing both parameters (percentage of ethylene glycol and concentration of Cyanex301) improves the extraction efficiency. The optimum percentage of ethylene glycol was equal to 80%.

The effect of cobalt concentration in the range of 600 to 1000 ppm in the aqueous solution was investigated. As shown in Fig. 4c, increasing both parameters (cobalt and Cyanex301 concentrations) improve the extraction efficiency due to more tendency for complex formation in the kerosene phase. The optimum concentration of cobalt ions was equal to 833 ppm.

The interaction between pH of an aqueous solution and ethylene glycol concentration is shown in Fig. 4d. It is observed that the increase and decrease behavior is shown in the extraction percentage of Co(II) removal with pH solution. The presence of ethylene glycol in the aqueous phase with the acidity of the aqueous solution showed the interaction due to the solubility of phases.

The 3D plot of cobalt concentration and pH of the aqueous system in Fig. 4e showed that the extraction increments with the tendency of reaction for complex formation. The more Co(II) concentration leads to lower efficiency due to the lower values of Cyanex301 extractant in the system (0.0275 M).

The effect of cobalt concentration in the range of 600 to 1000 and percentage of ethylene glycol in the range of 50 to 90 in kerosene is shown in Fig. 4f. It is observed that the lower change occurred under the variation of these parameters. The comparison of *F* values of the investigated parameters showed the more effect of Cyanex301 concentration on the extraction efficiency [Cyanex301 concentration (M) > pH solution > ethylene glycol (%v/v) > Co(II) concentration (ppm)] (see *F* values in Table 3).

#### Determination of optimum conditions

One of the best points in the experimental design was chosen with the optimization procedure with one desirability and maximum extraction efficiency. The optimal conditions were chosen as the concentration of Cyanex 301 (0.05 M), the concentration of cobalt (833 ppm), the pH (3.5), and the percent of EG (80%). The test was carried out in a 15 mL test tube, and the test extraction percentage matched that of the test design. The validation of data observed with the minimum errors between the predicted data (93.505%) and actual data (98.275%).

**Figure 5:**
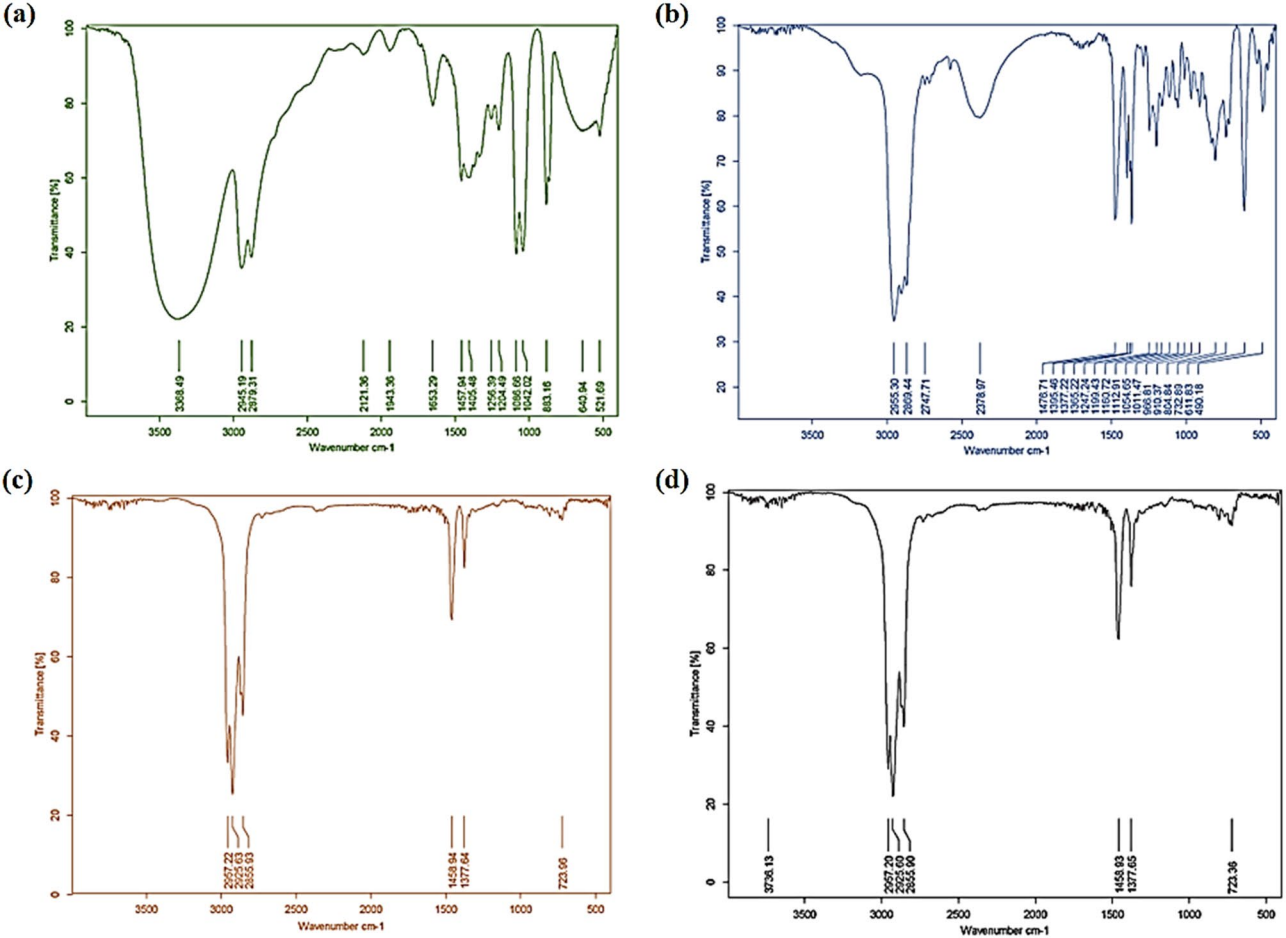
FTIR spectra of (**a**) pure ethylene glycol, (**b**) Cyanx301, (**c**) Cyanex301 + Co(II)/ extraction from aqueous system (%100 water) (**d**) Cyanex301 + Co(II)/ extraction from non-aqueous system (80% EG + 20%water (+ 0.1 M LiCl); 0.05 M Cyanex301; T = 25 °C; time = 10 min; Co(II) = 800 ppm; pH = 3.5).

Figure 5a,b,c,d showed the FT-IR spectra of pure ethylene glycol, pure Cyanex301, Cyanex301 + Co(II) from 100% aqueous phase, and Cyanex301 + Co(II) from 20% aqueous phase, 80% ethylene glycol, respectively. Characteristic vibrational bands from FTIR analysis is shown in Table 5. The S–H stretching peak of Cyanex301 is at 2378.97 cm^−1^ in Fig. 5b and disappears both in Fig. 5c,d because of the Cyanex301 − Co(II) complex formation. It indicates the replacement of the hydrogen in S–H by the metal ions. The absorption peak of P = S at 611.83 cm^−1^ in Fig. 5b shifts to 723.96 cm^−1^ in Fig. 5c,d,showing that there is a strong coordination effect between the P = S bond in Cyanex301 and the cobalt ions. The comparison of the organic phases in Fig. 5c,d showed the behavior of extraction in the non-aqueous system is similar to the aqueous system. At 2925 and 2957 cm^−1^, C–H traction bands are also visible. Because of the presence of CH_3_ group on the carbon atom. In EG spectrum (Fig. 5a), the OH group can be found around 3368 cm^-1^. The CH_2_ group vibration mode appears at 883 cm^−1^. The bands at 1042 and 1086 cm^−1^, respectively, indicate C–O and C–C stretching vibrations.

**Table 5:** Characteristic vibrational bands from FTIR analysis.

Bond	Wave number cm^−1^
P = S	611.83 for pure Cyanex301
	723.96 for Cyanex301 + Co(II) in SX system
	723.36 for Cyanex301 + Co(II) in NASX system
S – H	2378.97 for pure Cyanex301
C – H	2879.31, and 2945.19 for pure ethylene glycol
	2869.44, and 2955.30 for pure Cyanex301
	2925.63, and 2957.22 for Cyanex301 + Co(II) in SX system
	2925.60, and 2957.20 for Cyanex301 + Co(II) in NASX system
O – H	3368.49 for pure ethylene glycol
C – O	1042.02 for pure ethylene glycol
C – C	1086.66 for pure ethylene glycol

### Effect of temperature

**Figure 6:**
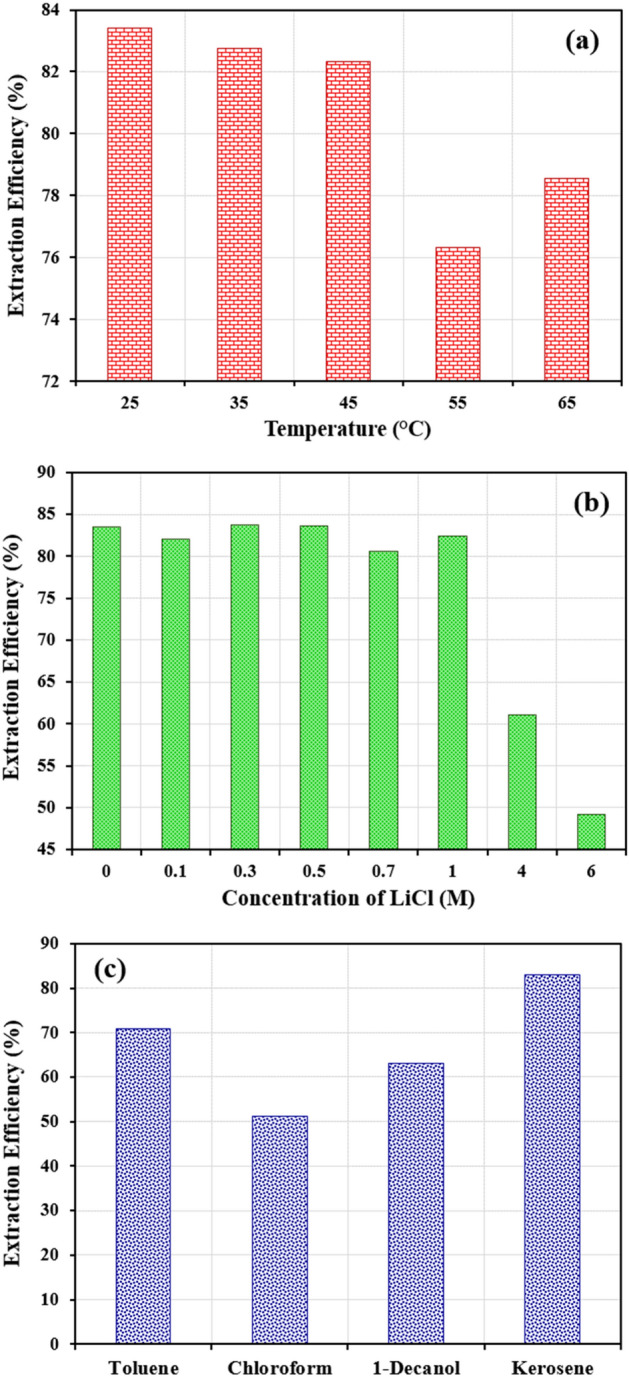
Effect of (**a**) temperature; (**b**) LiCl concentration; (**c**) type of solvent on cobalt extraction efficiency under optimum condition (80% EG; 0.05 M Cyanex301; time = 10 min; Co(II) = 800 ppm; pH = 3.5).

The effect of different temperatures (25, 35, 45, 55 and 65 °C) on cobalt extraction using Cyanex301 in kerosene in the optimum condition is shown in Fig. 6a. As can be seen, the increase in the temperature has a negligible effect on Co(II) extraction in the non-aqueous system. Therefore, the best temperature in this experiment is 25 °C (ambient temperature) or the same used in the experimental design.

### Effect of lithium chloride concentration

The addition of salts, especially lithium salts could largely enhance the immiscibility due to the salting-out effect of salts. Lithium salts are often selected because of their high solubility in many common polar molecular organic solvents^27^. The effect of changing the concentration of chloride ions in ethylene glycol solution from 0.1 to 6 M under optimum conditions is shown in Fig. 6b. Lithium chloride was selected as the chloride salt due to its high solubility in ethylene glycol. It is observed that more values for lithium chloride led to a diminish in cobalt removal in the non-aqueous system.

### Effect of different solvents

The effects of various solvents such as toluene, 1-decanol, chloroform and kerosene on cobalt extraction under optimum conditions is shown in Fig. 6c. In accordance with this table, it was observed that metals extraction efficiency increased with a decrease in the dielectric constant (ε) and the dipole moment (D). In high dielectric constants, a stronger interaction between the diluent and extractant was created and the amount of metal ion extraction decreased. As can be seen, the best solvents in this experiment are kerosene (ε:1.8; D:1.6) > toluene (ε:2.39; D:0) > 1-decanol (ε:8.1; D:1.6) > chloroform (ε:4.81; D:1.01), respectively.

### Phase ratios and McCabe diagram

**Figure 7:**
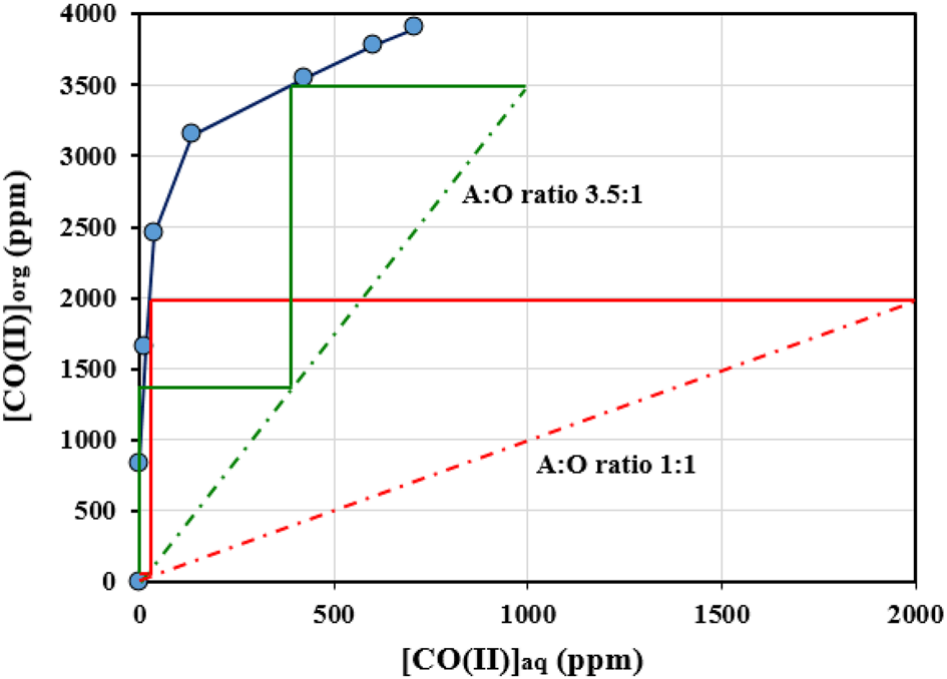
Cobalt extraction distribution isotherm and McCabe–Thiele diagram under optimum condition (80% EG (+ 0.1 M LiCl); 0.05 M Cyanex301; T = 25 °C; time = 10 min; Co(II) = 800 ppm; pH = 3.5).

It was feasible to compute the number of phases required for continuous extraction using a McCabe–Thiele diagram. This diagram for cobalt extraction employing phase ratios in Fig. 7 revealed that the one step in phase ratio 1:1 A/O and two steps in phase ratio 3.5:1 A/O are required to reach the maximum efficiency and the removal of cobalt ions.

### Stripping conditions

After performing the experiments and obtaining the extraction of cobalt ions to the organic phase, the different acids with various concentrations were used for the stripping condition. The results showed the sulphuric acid with 1 M concentration showed the maximum stripping percent equal to 95.43% in comparison to 1 M HCl solution (75.33%), and 1 M HNO_3_ solution (92.21%).

### Extraction equilibrium

**Figure 8:**
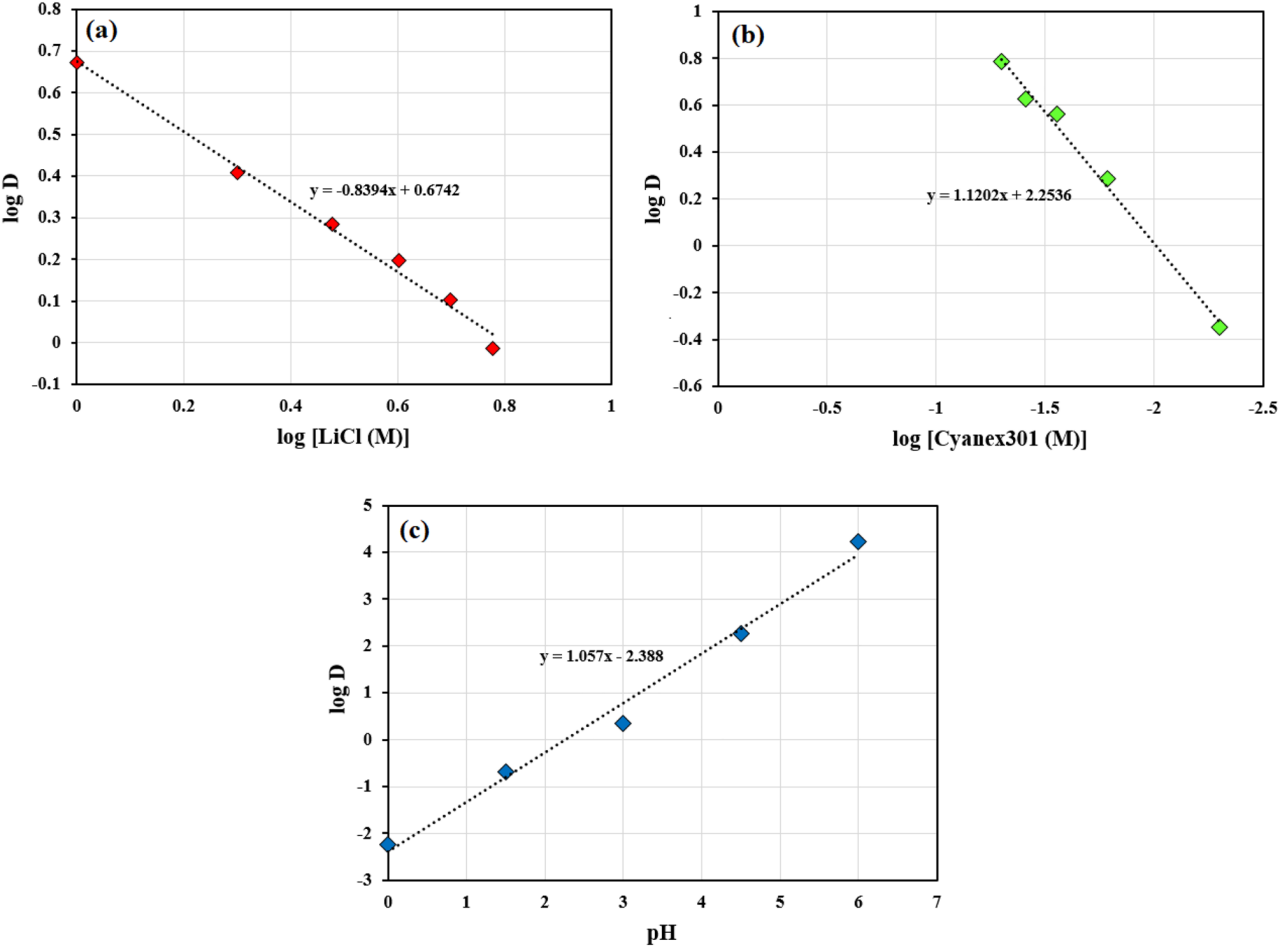
Effect of [LiCl (M)] concentration, Cyanex301 (M) concentration and aqueous pH on the distribution ratio under optimum condition (80% EG; T = 25 °C; time = 10 min; Co(II) = 800 ppm).

The cobalt ions are hydrated in aqueous solutions with six coordinated water molecules. The coordination chemistry of the hydrated cobalt(II) ions in the aqueous solution is [M(H_2_O)_6_]^2+^ complex. In the experimental conditions, the initial pH of Co(II) in the more polar phase is slightly acidic (pH ~ 6.0) which leads the ethylene glycol to give –OH group to Co(II). As shown in Fig. 8a, the distribution ratio slightly decreased with the increase in the anion concentration of LiCl. This indicates that no effect on the extraction of Co(II), the chloride ion wasn’t shared in the extracted complex accordingly. The plot of the distribution ratio versus the Cyanex301 concentration as a log–log plot in Fig. 8b is a straight line (slope 1.12). It was illustrated that one molecule of Cyanex301 in the complex formation. The variation of pH was carried out in the range from 0 to 6.0. As shown in Fig. 8c, the distribution ratio increments with the increase in the pH (slope 1.06). Therefore, one mole of protons from Cyanex301 transferred to the more polar phase for each formated complex.

Based on the slope analysis method, FTIR analysis, and the consideration of Cyanex301 in a dimeric acidic form in aliphatic diluents, the extraction of Co(II) through ethylene glycol (+ 1.0 mol/L LiCl) can be proposed over a cationic exchange reaction with one coordinated Cyanex301 molecules, as illustrated:

4 $$\left[ {Co\left( {OH} \right)\left( {H_{2} O} \right)_{x} \left( {EG} \right)_{y} } \right]^{ + } + \overline{{\left[ {\left( {HL} \right)_{2} } \right]}} \Leftrightarrow \overline{{\left[ {Co\left( {OH} \right)L.HL} \right]}} + H^{ + } + xH_{2} O + yEG$$

In the above equation, EG, (HL)_2_ are ethylene glycol, the dimeric form of Cyanex301 in kerosene, respectively. The bars refer to the less polar phase species and x, y measure to the stoichiometric number of water and ethylene glycol molecules coordinating to Co(II) in the more polar phase, respectively.

### Extraction from leaching solution

**Figure 9:**
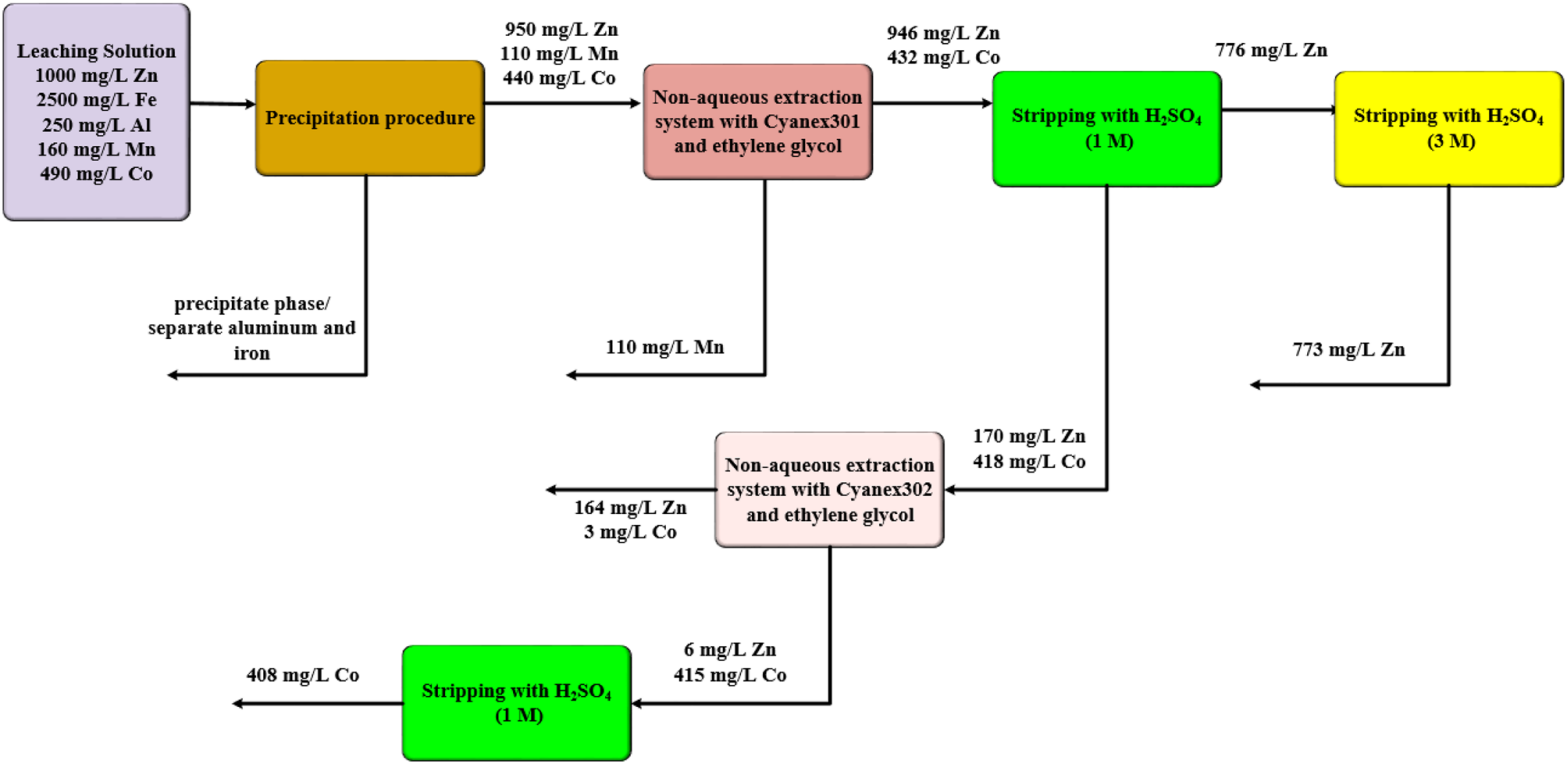
Extraction and recovery of cobalt ions from zinc plant residue.

In this study, the real solution was prepared with acid leaching step from zinc plant residue. The leach solution consists of cobalt 490 ppm, zinc 1000 ppm, manganese 160 ppm, aluminum 250 ppm, and iron 2500 ppm. The iron, and aluminum were precipitated with NaOH solution in pH ~ 4 and their concentrations were lower than one ppm. Then, the leach solution containing zinc, cobalt and manganese was used in the non-aqueous system to investigate the extraction efficiency in the presence of impurities. Cyanex301 extractant (0.05 M) diluted in kerosene was used as the organic phase. The non-aqueous system including 80% ethylene glycol, 0.1 M LiCl, pH ~ 3.5 and 20% leach solution (Zn(II), Mn(II), and Co(II)) was used in the experiments, according to the optimum condition. The procedure of extraction is shown in Fig. 9. The zinc and cobalt ions were transferred to the organic phase. But, the manganse ions was remained in the leaching solution. The third step was used for the recovery of cobalt ions from the organic phase with the sulphuric acid solution (1 M) as the stripping stage. In this step, cobalt ions were transferred to the stripping phase. Also, some of zinc ions was transferred in the stripping phase. but, the more zinc ions was separated using 3 M sulphuric acid solution in the fourth step from the same organic phase. The separation of zinc and cobalt in the third step was carried out with the contact of stripping phase with organic phase containing Cyanex302 diluted in kerosene. The results of Rashchi and co-workers reported that Cyanex302 as the best extractant for the separation of zinc and cobalt ions^36^. Cyanex302 extractant (0.05 M) diluted in kerosene was used as the organic phase. The non-aqueous system including 80% ethylene glycol, 0.1 M LiCl, and 20% stripping solution (Zn(II), and Co(II)) was used. The results showed that the separation of cobalt, zinc and manganese ions was obtained with this procedure.

## Conclusions

The polarity and hydrophobicity of the two phases are the most significant aspects in creating non-aqueous solvent extraction systems (NASX). They are constructed by selecting the appropriate more polar and less polar phases to form immiscible two-phase systems. NASX adds a new dimension to metal separations by allowing various metals to respond differently in terms of speciation in different solvents and at variable anion and water concentrations. In this study, a new extraction method using a non-aqueous solvent extraction strategy with the central composite design approach was examined for cobalt extraction from zinc plant residue. According to the findings, the best extractant from ethylene glycol solution (+ 0.1 M LiCl) was Cyanex301. The desalination agent was chosen at 0.1 M lithium chloride, which has little influence on cobalt extraction. In the metallurgical solution procedure, kerosene was also found to be the optimum solvent. Extraction efficiency improves by raising the concentration of the Cyanex301 extractant, and this parameter showed the main effect on cobalt removal. Increasing the temperature does not influence cobalt extraction from ethylene glycol solution, according to the results of the experiments. The findings of this investigation revealed that non-aqueous systems containing ethylene glycol can be used a substitute for aqueous systems with reasonable success for the extraction from leaching solution containing various metal ions.

## Data Availability

The datasets used and/or analyzed during the current study are available from the corresponding author on reasonable request.
